# Pectinesterase Inhibitor from Jelly Fig (*Ficus awkeotsang* Makino) Achene Inhibits Surface Antigen Expression by Human Hepatitis B Virus

**DOI:** 10.1155/2013/434823

**Published:** 2013-11-04

**Authors:** Yu-Chuen Huang, Chii-Ming Jiang, Yu-Jen Chen, Yu-Yawn Chen

**Affiliations:** ^1^Genetics Center, Department of Medical Research, China Medical University Hospital, Taichung 404, Taiwan; ^2^School of Chinese Medicine, China Medical University, Taichung 404, Taiwan; ^3^Graduate Institute of Biostatistics, China Medical University, Taichung 404, Taiwan; ^4^Department of Food Science and Technology, National Taitung Junior College, Taitung 950, Taiwan; ^5^Department of Seafood Science, National Kaohsiung Marine University, Kaohsiung 811, Taiwan; ^6^Department of Radiation Oncology, Mackay Memorial Hospital, 92 Chung San North Road, Section 2, Taipei 104, Taiwan; ^7^Institute of Traditional Medicine, National Yang-Ming University, Taipei 112, Taiwan; ^8^Department and Graduate School of Physical Education, National Taiwan University of Physical Education and Sport, Taichung 404, Taiwan

## Abstract

Pectinesterase inhibitor (PEI) isolated from jelly fig (*Ficus awkeotsang* Makino) is an edible component of a popular drink consumed in Asia. Hepatitis B virus (HBV) infection is prevalent in Asia, and current treatments for HBV infection need improvement. This study aimed to evaluate the effect of PEI on the surface antigen expression by HBV (HBsAg). Human hepatoma cell lines Hep3B and Huh7 served as in vitro models for assessing the cytotoxicity and HBsAg expression. A culture of primary hepatocytes cultured from mice served as the normal counterpart. Cell viability was measured by 3-(4,5-dimethylthiazol-2-yl)-2,5-diphenyltetrazolium bromide (MTT) colorimetric assay. HBsAg expression was evaluated by measuring HBsAg secretion into the culture medium using an enzyme-linked immunosorbent assay. The results showed that PEI did not affect the viability of the human hepatoma cell lines or primary mouse hepatocytes. PEI inhibited the expression of HBsAg in hepatoma cell lines harboring endogenous (Hep3B) and integrated (Huh7) HBV genomes in a concentration- and time-dependent manner, thus implicating a universal activity against HBV gene expression. In conclusion, it suggests that PEI from jelly fig inhibits the expression of human HBsAg in host cells without toxic effects on normal primary hepatocytes.

## 1. Introduction

Hepatitis B virus (HBV) infection is an important health issue because of its increasing prevalence. It is highly correlated with the development of cirrhosis and development of hepatocellular carcinoma, a malignancy with an extremely poor prognosis. The current antiviral agents used to treat HBV infection include lamivudine, adefovir, entecavir, and interferon- (IFN-) *α* [1]. However, the effectiveness of antiviral agents, particularly nucleoside analogs, is limited by drug resistance due to gene mutations in HBV caused by long-term administration [2, 3]. For example, lamivudine administration causes high rates of resistance due to emergence of HBV strains with the YMDD mutation [4]. Therapy with IFN-*α* for a finite duration is often interrupted by its toxic effects such as pyrexia, fatigue, headache, myalgia, depression, and myelosuppression [5]. These limitations compromise the therapeutic efficacy of current treatments for HBV infections. Clearly, it is important to develop a novel category of therapeutics for HBV infection based on an innovative strategy.

Jelly fig (*Ficus awkeotsang* Makino) is a native woody vine that grows extensively in Taiwan. A jelly curd produced from the water extract of the jelly fig achenes in the presence of calcium is a popular drink. Pectinesterase (PE) catalyzes the deesterification of pectin and converts the protopectin into soluble pectin and pectate. Previously, Jiang et al. reported that PE activity in a solution derived from intact achenes gradually increased and then decreased to almost zero during the 90 h extraction of PE [6]. Intriguingly, the PE activity of jelly fig achenes was eliminated when a homogenized solution of crushed achenes was used with the same PE extraction procedure. Therefore, some substances released from crushed achenes during PE extraction, that is, PE inhibitors (PEIs), inhibit the PE activity and decrease the PE-catalyzed reaction rate [6]. PEIs prepared from intact jelly fig achenes were previously separated and characterized as polypeptides with molecular weights of 3.5–4.5 kDa, comprising more than 50% histidine. These PEIs displayed marked competitive inhibition of the activity of PEs derived from fruits and vegetables [6]. The known biological activity of PEI includes growth inhibition in human leukemic U937 cells by inducing cell cycle arrest and apoptosis through intrinsic pathway involvement and caspase 3 activation [7]. However, the bioactivity of PEIs against viruses remains unknown.

This study aimed to examine the anti-HBV effect of a natural dietary constituent purified from jelly fig. Surface antigen expression by HBV in host cells served as the major endpoint. Primary hepatocytes isolated from mice were used as the normal counterpart to assess safety. 

## 2. Materials and Methods

### 2.1. Preparation of PEI from Jelly Fig Achenes

 The PEI solution was prepared from pectin-depleted jelly fig achenes by the Department of Seafood Science, National Kaohsiung Marine University, Kaohsiung, Taiwan, as reported previously [6]. The yield of PEI extraction from jelly fig achenes was around 0.1% (w/w). In brief, achenes were rinsed in 20 volumes (w/v) of 5% NaCl solution repeatedly to wash out the major pectin and PE; subsequently, they were homogenized using a blender (6000 rpm) for 2 min. After being extracted using 15 parts (1/15, w/v) of distilled water for 5 h and centrifuged (10000 ×g, 10 min, 4°C), the supernatant was heated in a boiling water bath for 30 min to denature the residual PE. The crude PEI solution was concentrated using a rotary evaporator under reduced pressure (<100 mm Hg). The concentrates were extracted using 250 mL of ethyl acetate, and this was repeated four times. These concentrates (1000 mL) were evaporated under reduced pressure (<100 mm Hg) at room temperature to obtain the PEI concentrate. Further separations were performed by loading approximately 300 mg of the PEI concentrate with ethyl acetate into a 25 × 400 mm glass column packed with ToyoPearl HW-40S resin (Tosoh Bioscience LLC, Tokyo, Japan), by monitoring at 280 nm. Methanol elution [methanol : acetone : water (45 : 25 : 30, v/v/v)] at a flow rate of 1.5 mL/min was used to separate the PEI concentrate. The fourth pooled fractions with PE inhibitory activity (termed as PEI) were lyophilized and stored at −20°C until use. Cells were incubated in 35 mm Petri dishes at an initial concentration of 10^5^ cells/mL in the presence of PEI (50–400 *μ*g/mL) at 37°C in a humidified 5% CO_2_ incubator.

### 2.2. Hepatoma Cells and Culture

Human hepatocellular carcinoma cell lines Hep3B and Huh7 cells harbor endogenous and integrated HBV genomes, respectively, with stable production of HBsAg and served as in vitro models of HBV replication [8, 9]. These hepatoma cells were cultured in Dulbecco's modified Eagle's medium (DMEM) containing 10% fetal calf serum, 10^5^ I.U./L penicillin, 100 mg/L streptomycin, and 1,000 micromole/L L-glutamine in a humidified 5% CO_2_ incubator at 37°C. 

### 2.3. Preparation and Culture of Primary Mouse Hepatocytes

Primary hepatocytes were isolated from 6- to 8-week-old male BALB/c mice using the procedures described by Klaunig et al. with some modifications [10]. After anaesthetization and laparotomy, the portal vein was cannulated and perfused with calcium-free Hanks' balanced salt solution (HBSS) containing ethylene glycol-bis-(b-aminoethyl) N, N9-tetraacetic acid, and N-2-hydroxyethylpiperazine-N-2-ethane sulfonic acid. This was followed by perfusion with HBSS containing collagenase for isolation of hepatocytes. After removing the liver, the hepatocytes were mechanically dissociated and filtered through a mesh and were isolated by gradient centrifugation with Percoll (GE Healthcare, Little Chalfont, UK) and washed. Cells were cultured further and plated with complete medium and adequate hepatocyte growth factor. The medium was changed regularly or as required.

### 2.4. Growth Inhibition and Cell Viability Assessment Using the MTT Assay

The viability of hepatoma cells was assessed using a tetrazolium dye colorimetric MTT [3-(4,5-dimethylthiazol-2-yl)2,5-diphenyl tetrazolium bromide] assay [11] and expressed as the MTT value of the experimental group divided by the MTT value of the untreated control group. 

### 2.5. Assay to Determine Relative HBsAg Expression

Hepatoma cells were cultured in DMEM with 10% fetal bovine serum for 24 h and transferred to serum-free DMEM with or without PEI and incubated thereafter. The HBsAg secreted in the culture medium was measured using a commercial enzyme-linked immunosorbent assay (ELISA) kit (General Biological, Taipei, Taiwan). The optical density values determined using ELISA were normalized with the cell viability. The detection of HBsAg in the sera of patients indicates an existing HBV infection and the risk of developing cirrhosis and hepatocellular carcinoma. Thus, HBsAg is a useful index for evaluating viral activity [12]. The relative HBsAg expression was determined using the following formula: (HBsAg/MTT) from PEI-treated cells/(HBsAg/MTT) from the untreated cells. (HBsAg/MTT) from the untreated control group was treated as a 100% expression. Measurement of viral antigen secretion by HBV host cells, such as HBsAg secretion by Hep3B cells, has been used in various studies to evaluate the effects of cytokine drugs on HBV.

### 2.6. Statistical Analysis

 Results are presented as mean ± SEM. Differences among the treatment groups were assessed by Student's *t*-test or ANOVA test. Statistical analyses were performed using the Statistical Package for the Social Sciences software package, v18.0 (SPSS Inc., Chicago, IL), and a *P* value of <0.05 was considered significant.

## 3. Results

### 3.1. Effect of PEI on the Viability of Hepatoma Cells

**Figure 1:**
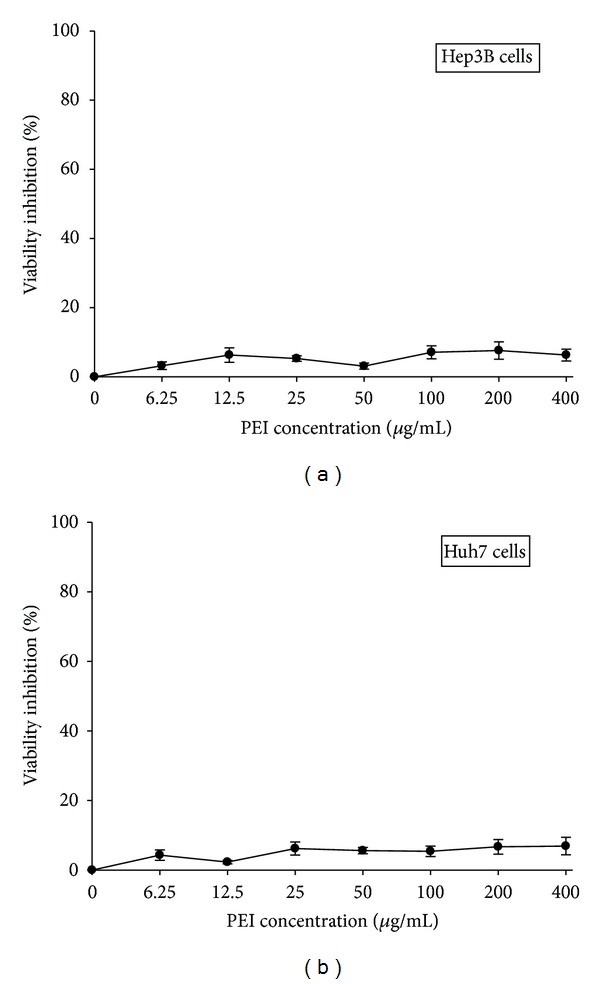
The viability of (a) Hep3B and (b) Huh7 human hepatoma cells with PEI treatment. Cells were treated with various concentrations of PEI for 48 h and then subjected to MTT assay. Triplicated data from separate experiments are expressed as mean ± SEM.

 To determine the cytotoxicity of PEI on the human hepatoma cells, Hep3B and Huh7 cells were treated with various concentrations of PEI (0, 6.25, 12.5, 25, 50, 100, 200, and 400 *μ*g/mL) for 48 h and then subjected to MTT assay. As shown in Figures 1(a) and 1(b), PEI at concentrations of 0–400 *μ*g/mL did not significantly affect the cell viability of Hep3B and Huh7 cells, respectively. Therefore, the biological activity of PEI in these cells was probably not due to cellular toxicity. 

### 3.2. Reduction of the Relative HBsAg Expression in Human Hepatoma Cells by PEI

**Figure 2:**
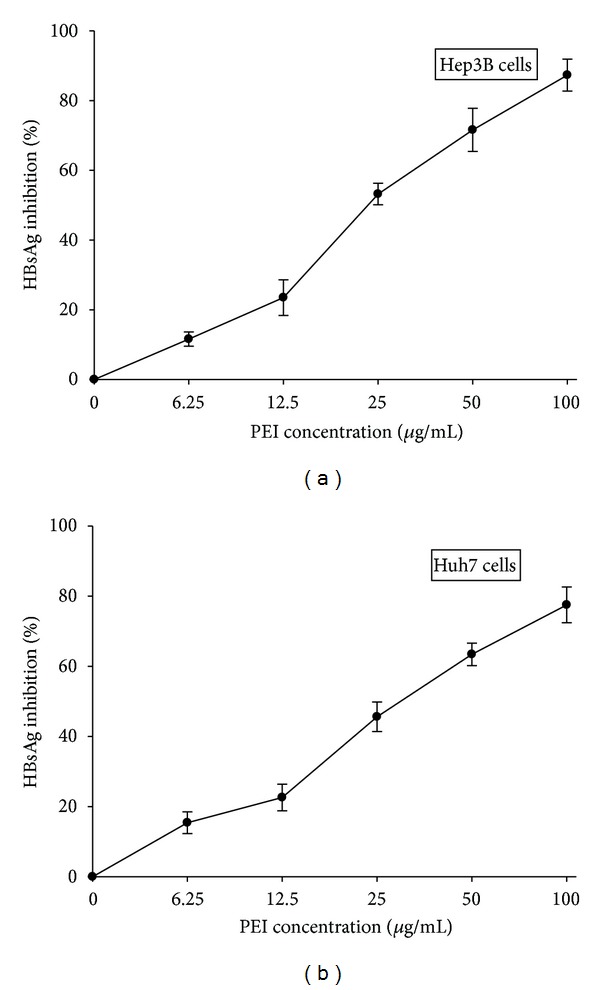
The relative HBsAg expression of (a) Hep3B and (b) Huh7 human hepatoma cells treated with various concentrations of PEI for 48 h. Triplicated data from separate experiments are expressed as mean ± SEM.

**Figure 3:**
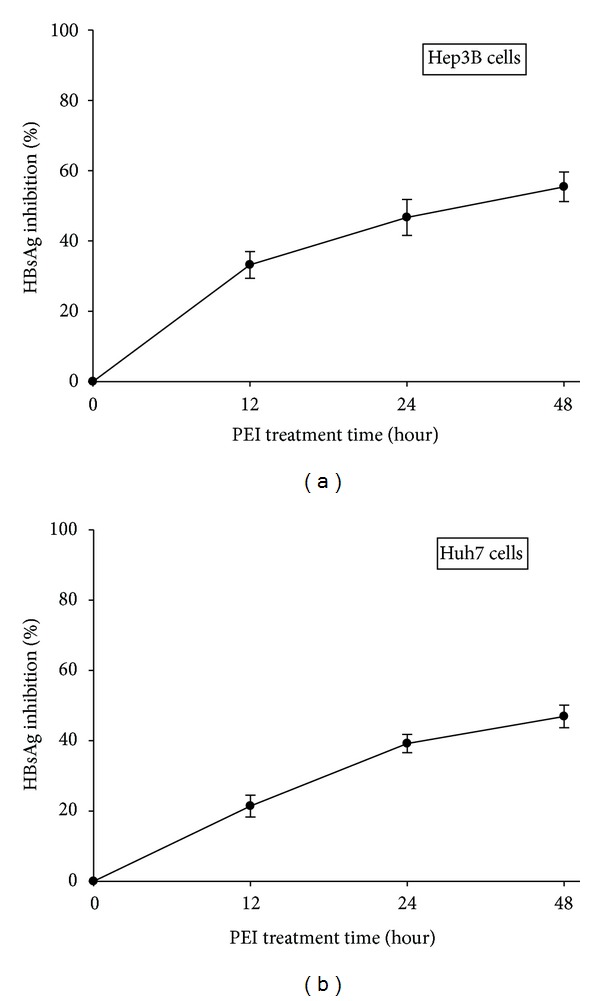
The relative HBsAg expression of (a) Hep3B and (b) Huh7 human hepatoma cells at 25 *μ*g/mL of PEI for 12, 24, and 48 h. Triplicated data from separate experiments are expressed as mean ± SEM.

To examine the relative expression levels of HBsAg in human hepatoma cells exposed to PEI. Hep3B and Huh7 cells were treated with different concentrations of PEI (0, 6.25, 12.5, 25, 50, and 100 *μ*g/mL) for 48 h in serum-free medium after serum starvation. The culture medium was then harvested and the amount of HBsAg was measured by ELISA. As shown in Figures 2(a) and 2(b), PEI suppressed the endogenously expressed HBsAg in Hep3B cells and suppressed the HBsAg produced from the stable clone of HBV DNA-integrated in human hepatoma Huh7 cells without endogenous HBV genomes. This inhibitory effect was concentration-dependent with 87.3% ± 4.6% and 77.5% ± 5.1% reductions at 100 *μ*g/mL of PEI in Hep3B and Huh7 cells, respectively (Figures 2(a) and 2(b)). In addition, at the 50% HBsAg-inhibitory concentration, that is, 25 *μ*g/mL of PEI, a time-dependent effect (12, 24, and 48 h) was also demonstrated in Hep3B and Huh7 cells, as shown in Figures 3(a) and 3(b).

### 3.3. Effect of PEI on the Viability of Normal Hepatocytes

**Figure 4:**
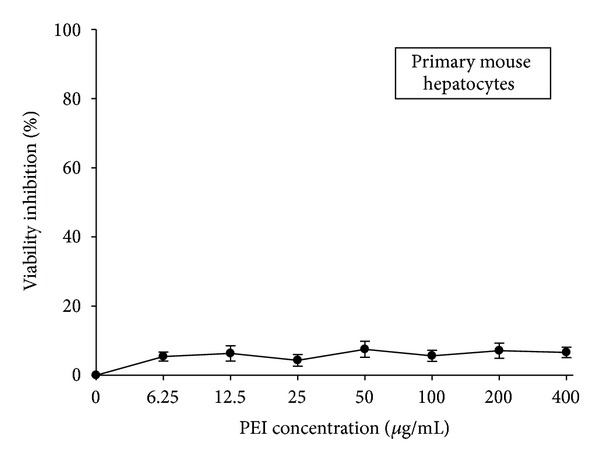
The viability of primary cultured hepatocytes with PEI treatment. Cells were treated with various concentrations of PEI for 48 h and then subjected to MTT assay. Triplicated data from separate experiments are expressed as mean ± SEM.

 Primary cultured hepatocytes isolated from BALB/c mice served as a normal counterpart for assessment of PEI toxicity in normal tissue. Normal hepatocytes were treated with various concentrations of PEI (0, 6.25, 12.5, 25, 50, 100, 200, and 400 *μ*g/mL) for 48 h and then subjected to MTT assay. As shown in Figure 4, PEI at concentrations greater than those that effectively inhibited HBsAg expression in hepatoma cells did not significantly decrease the viability of normal hepatocytes.

## 4. Discussion

We found that PEI isolated from jelly fig, a raw material found in a popular drink in Asia, inhibited the expression of human HBsAg in host cells without toxic effects on normal primary hepatocytes. 

 As mentioned previously, the current treatments for HBV are either toxic or have a tendency of resulting in resistance through gene mutations. We found that the naturally occurring PEI from jelly fig probably inhibits HBsAg expression without any toxicity to hepatocytes, which may facilitate the development of novel therapeutic agents. PEI from jelly fig comprises thermally stable polypeptides (3.5–4.5 kDa) with 57% basic amino acids, which inhibited the intrinsic carambola PE activity required for methanol reduction in wines [13]. It is unknown whether the bioactivity of PEI from jelly fig will be retained in the gastrointestinal tract after digestion. Thus, further in vivo tests are required. 

 The experimental models tested in this study comprised Hep3B cells that harbored endogenous HBsAg and a stable clone of HBV DNA-integrated Huh7 cells. PEI inhibited HBsAg expression in both cell lines, indicating its universal activity against HBsAg expression. However, this experimental model only provides a screening platform for anti-HBV agents [14]. Further molecular studies, including viral replication assays, are required to confirm whether PEI from jelly fig can inhibit HBV. 

 Normal liver toxicity assessment based on the in vitro hepatocyte viability showed no evidence of PEI hepatotoxicity. Currently used anti-HBV agents have various levels of toxicity [5], which further supports the potential development of PEI as a novel anti-HBV agent. 

 In clinical practice, chemotherapy-related HBV reactivation and subsequent fulminant failure is not rare in HBV-infected patients [15, 16]. The prophylactic administration of antiviral agents such as lamivudine has been shown to be effective [17]. Given that PEI is found in a safe and commonly used beverage, it is expected that PEI may be another option for prophylaxis against HBV reactivation in patients with cancer who already receive many chemotherapy agents.

## 5. Conclusions

In conclusion, PEI isolated from jelly fig may inhibit human HBsAg expression in host cells without any toxic effects on normal hepatocytes.
